# Protective Effects of Panax notoginseng Saponins on Cerebral Ischemia/Reperfusion Injury: Insights Into SIRT1/NRF2/HO-1 Pathway Activation

**DOI:** 10.33549/physiolres.935458

**Published:** 2025-04-01

**Authors:** Jun WU, Xi CHEN, Xiwen LIN, Zhe LI, Zhiwei CAO, Wenbiao HUANG, Dongchuan SHAO, Shaik Althaf HUSSAIN, Kuairong PU, Nan ZHAO

**Affiliations:** 1 Department of Neurosurgery, The First Hospital of Kunming (Affiliated Calmette Hospital of Kunming Medical University), Kunming, China; 2 Department of Zoology, College of Science, King Saud University, Riyadh, Saudi Arabia

**Keywords:** Ischemia/reperfusion injury, Neuroprotection, Oxidative stress, Panax notoginseng saponins, Stroke

## Abstract

Stroke and cerebral ischemia/reperfusion (IR) injury are severe conditions characterized by impaired blood flow to the brain, leading to tissue infarction and neurological impairments. Panax notoginseng saponins (PNS) have displayed various beneficial effects in alleviating cerebrovascular disorders. This study aimed to investigate the neuroprotective capacity of PNS in a rat model of middle cerebral artery occlusion (MCAO)-induced cerebral IR injury, focusing specifically on understanding the involvement of the sirtuin 1 (SIRT1)/nuclear factor erythroid 2-related factor 2 (Nrf2)/heme oxygenase-1 (HO-1) pathway in mediating this protective effect. Male Sprague-Dawley rats (n=45, weighing 250–280g and aged 12 weeks) were utilized in this experiment. Cerebral IR injury was induced by subjecting the rats to 30 minutes of MCAO followed by 24 hours of reperfusion. Prior to the surgery, PNS (120mg/kg) was administered once daily *via* gavage for 14 days. The evaluation measures included assessing cerebral infarct volume, neurological function using the Longa method, conducting histopathological analysis, examining the expression of SIRT1, Nrf2, and HO-1 genes and proteins, as well as measuring the levels of glutathione (GSH), superoxide dismutase (SOD), and malondialdehyde (MDA). Pretreatment with PNS markedly decreased infarct volume, enhanced neurological function, and mitigated histopathological alterations. Additionally, PNS intake resulted in the upregulation of SIRT1, Nrf2, and HO-1 genes and proteins, boosted enzymatic antioxidant activity, and lowered MDA levels, pointing towards a diminution in oxidative stress. The multifaceted antioxidant and neuroprotective properties of PNS underscore its promising role in preserving neuronal function, mitigating oxidative damage, and promoting tissue survival in ischemic conditions. These benefits were associated with the modulation of the SIRT1/Nrf2/HO-1 signaling pathway, emphasizing the therapeutic significance of PNS in addressing cerebral IR injury and related neurological complications.

## Introduction

Cerebral ischemia/reperfusion (IR) injury arises from the interruption and subsequent restoration of blood flow to the brain. This condition, often linked to stroke, can result in substantial brain damage and neurological impairments [1]. During the initial ischemic phase, brain tissue experiences deprivation of oxygen and essential nutrients, leading to energy depletion, cell demise, and the accumulation of harmful metabolites. Paradoxically, the following reperfusion phase, crucial for reinstating blood circulation, worsens brain injury through intricate biochemical and molecular processes [2]. Current therapeutic approaches for cerebral IR injury are restricted and mainly concentrate on reperfusion methods like thrombolysis and mechanical thrombectomy, effective only within a narrow post-stroke timeframe. These treatments inadequately address secondary injury mechanisms such as oxidative stress and inflammation, which persist in harming brain tissue even after reperfusion [3]. Hence, there is a pressing demand for innovative therapeutic strategies targeting these secondary injury pathways. Natural compounds, with their encouraging preclinical outcomes, offer a promising avenue for such research, providing optimism for more efficient and comprehensive stroke treatments [4,5].

The pathophysiology of cerebral IR injury involves an intricate interplay of oxidative stress, inflammation, and cell demise [6]. During ischemia, the absence of oxygen and essential nutrients leads to energy depletion and accumulation of metabolic byproducts. When blood flow is restored, the sudden influx of oxygen results in the generation of reactive oxygen species (ROS). These highly reactive molecules cause extensive damage to cellular components such as lipids, proteins, and DNA, setting off oxidative stress. This oxidative damage is a critical factor in the secondary injury cascade, which persists in damaging brain cells beyond the ischemic episode [7, 8]. One of the vital pathways involved in the cellular response to oxidative stress is the sirtuin 1 (SIRT1)/nuclear factor erythroid 2-related factor 2 (Nrf2)/heme oxygenase-1 (HO-1) signaling pathway [9]. In the context of cerebral IR injury, SIRT1 deacetylates and activates various transcription factors that boost the production of antioxidant enzymes. Additionally, it stabilizes Nrf2, a chief regulator of the antioxidant response. Upon activation, Nrf2 moves to the nucleus and binds to antioxidant response elements in the DNA, stimulating the expression of numerous antioxidant genes. Activation of Nrf2 results in the increased expression of HO-1, an enzyme that breaks down heme into biliverdin, free iron, and carbon monoxide. Biliverdin and its product, bilirubin, are potent antioxidants that aid in neutralizing ROS and lessening oxidative damage [10–12]. The SIRT1/Nrf2/HO-1 pathway offers a robust neuroprotective mechanism that can substantially enhance outcomes for stroke patients. Targeting this pathway could lead to the development of innovative neuroprotective approaches, providing optimism for enhanced recovery and reduced long-term disability [13].

Panax notoginseng, commonly known as Sanqi or Tianqi, is a traditional herbal product commonly utilized in Chinese medicine [14]. The main active components of Panax notoginseng are saponins, known as Panax notoginseng saponins (PNS), which have attracted considerable interest due to their diverse pharmacological properties and therapeutic benefits [15]. The therapeutic potential of PNS has been demonstrated in preclinical models of various conditions, including nervous system disorders [16,17], diseases [18,19], diabetes [20], and specific types of cancer [21–23]. PNS exhibit significant neuroprotective effects by reducing neuronal cell death and promoting neuronal viability [24]. They boost the body’s natural antioxidant defenses by enhancing the activity of enzymes like glutathione (GSH), glutathione peroxidase, superoxide dismutase (SOD), and catalase, thereby defending cells against damage and supporting overall cellular health [25,26]. Additionally, PNS regulate inflammatory processes by inhibiting the production of pro-inflammatory cytokines and suppressing inflammatory pathways’ activation [27,28]. The multifaceted ability of PNS to influence several biological pathways highlights their potential as multi-target therapeutic agents within the realm of traditional Chinese medicine. Ongoing research continues to explore the broad therapeutic potential of PNS, providing a foundation for their possible role as complementary interventions in conjunction with contemporary evidence-based medical practices [29].

In this study, we employed a preclinical model of cerebral IR injury in rats to assess the neuroprotective properties of PNS. By utilizing an animal model that closely mimics the pathophysiology of human stroke and conducting a comprehensive evaluation of both biochemical and functional parameters, our research offers valuable insights into the potential application of PNS as a therapeutic approach for stroke patients. While previous studies have explored the neuroprotective effects of PNS, this research uniquely delves into the molecular mechanisms by which PNS exert their neuroprotective actions, particularly by modulating the SIRT1/Nrf2 pathway. Understanding these underlying mechanisms is essential for developing targeted therapeutic interventions that can effectively mitigate oxidative stress and minimize brain damage in stroke patients.

## Methods

### Animal subjects

In this research, forty-five male Sprague-Dawley (SD) rats, each weighing between 250 to 280 grams and aged 12 weeks, were utilized. The rats were housed in conventional laboratory conditions, with temperature maintained within the range of 20 to 24 °C and a humidity level of 55 %. They were subjected to a 12-hour light-dark cycle and had continuous access to food and water. The handling and experimental procedures carried out on the animals followed the guidelines outlined by the National Institutes of Health for the ethical treatment of research animals (8th Edition, revised 2011). Furthermore, all protocols were implemented in accordance with the ethical standards and regulations set by the Animal Experiment Ethics Review Committee of Kunming Medical University (No. kmmu20230131).

### Panax notoginseng saponins (PNS) drug

PNS was sourced from Chengdu Manster Biotechnology Co., Ltd. (DST200706-054; Chengdu, China). The major active constituents of PNS included 6.96 % Notoginsenoside R1, 34.29 % Ginsenoside Rg1, 2.65 % Ginsenoside Re, 41.34 % Ginsenoside Rb1, and 10.19 % Ginsenoside Rd, measured by volume. A fresh PNS solution was prepared daily using 0.5 % sodium carboxymethyl cellulose, purchased from Dalian Meilun Biotechnology Company (Dalian, China).

### Group assignment and treatment protocol

To investigate the impact of PNS on stroke outcomes, a total of forty-five male SD rats were randomly divided into three distinct experimental groups, each comprising 15 rats. The allocation of this specific number was based on meticulous statistical power analysis to ensure the credibility and robustness of the ensuing results. In a measure to mitigate potential bias, a random allocation strategy was implemented, and the evaluators tasked with assessments were blinded to the group allocations, fostering an impartial and unbiased evaluation process. The categorization of the groups was structured as follows:

- Sham: Rats in this group underwent the surgical intervention devoid of middle cerebral artery occlusion (MCAO) initiation or PNS administration;
- MCAO: This group of rats underwent MCAO surgery to induce cerebral IR injury but did not receive any PNS intervention; and
- MCAO + PNS: Rats in this subset were subjected to a daily gavage regimen of 120 mg/kg PNS, for a duration of 14 days preceding the surgical procedure [30].

Both the Sham and MCAO groups received an equivalent volume of the vehicle via oral gavage. After 24 hours post-surgery, five rats from each group were chosen for the assessment of cerebral infarct volume. Additionally, another batch of 5 rats per group underwent neurological evaluation to observe their behavioral responses. Following the evaluation, the rats were euthanized under deep anesthesia in accordance with ethical guidelines, and their brain tissues were harvested for histological examination to investigate potential cellular alterations. Finally, the brains of the remaining 5 rats in each group were utilized for molecular and biochemical analyses, focusing on changes in gene expression, protein levels, and markers of oxidative stress.

### Establishment of MCAO model

To induce cerebral IR injury in rats, resembling the pathophysiology of a stroke, the MCAO model was utilized. The surgical intervention was conducted under sterile conditions with meticulous care to minimize potential complications. Initially, rats were anesthetized via intraperitoneal administration of 50 mg/kg pentobarbital sodium. Subsequent to anesthesia, rats were placed in a supine position on a temperature-regulated heating pad to maintain consistent body warmth throughout the procedure. The cervical region was shaved and disinfected using an antiseptic solution. A midline neck incision was made to expose the right common carotid artery, external carotid artery, and internal carotid artery. Delicately separating the connective tissues and muscles facilitated a clear view of the carotid arteries. Subsequently, the external carotid artery was ligated, and a nylon monofilament with a silicone-coated tip was passed through the external carotid artery into the internal carotid artery until resistance was encountered. The monofilament was retained for a period of 30 minutes to induce cerebral ischemia, following which it was gently retracted to initiate reperfusion. Rats in the Sham group underwent an identical surgical protocol sans filament insertion. Post-surgery, the incision site was meticulously sutured, and the rats recuperated in a warm, tranquil, and controlled setting. They were then reintroduced to their housing quarters and meticulously monitored during the postoperative phase.

### Measurement of cerebral infarct volume

Following 24 hours of reperfusion, each group of five rats underwent deep anesthesia induced by intraperitoneal injection of 50 mg/kg pentobarbital sodium. Subsequent transcardial perfusion was carried out using heparinized saline, followed by 4 % paraformaldehyde (Sigma-Aldrich, Saint Louis, MO, USA). The brains were meticulously extracted from the cranial cavity, post-fixed in 4 % paraformaldehyde for 24 hours, and then immersed in a 30 % sucrose solution for cryoprotection. These frozen brains were sectioned coronally on a cryostat, generating slices with a thickness of 20 μm. Sequential sections were collected at predefined intervals spanning the infarcted area. The brain sections were then immersed in a 2 % solution of 2,3,5-triphenyltetrazolium chloride (TTC) (Sigma-Aldrich, Saint Louis, MO, USA) in phosphate-buffered saline (PBS) at 37 °C for 30 minutes. Post-incubation, the sections were rinsed with PBS to eliminate excess TTC solution, mounted on glass slides, and covered with a mounting medium. Utilizing a bright-field microscope equipped with a digital camera, digital images of the stained brain sections were captured. The infarct region (displaying a pale, unstained appearance) and the corresponding hemisphere were manually delineated using image analysis software. The infarcted region was quantified through planimetry methods, where the areas from all sections were totaled and then multiplied by the section thickness to derive the infarct volume.

### Neurological assessment

Following a 24-hour reperfusion period, each group of rats (n=5) underwent neurological assessment based on the Longa method. The grading scale used was as follows: 0=absence of neurological deficits; 1=inability to extend the left forelimb while lifting the tail; 2=circling towards the opposite side when ambulating; 3=leaning towards the opposite side while walking; 4=absence of spontaneous motor activity or unconsciousness [31]. Subsequently, at the conclusion of the study, rats were euthanized under deep anesthesia, and their brain tissues were harvested for further histological parameter analysis.

### Histological analysis

After assessing the neurological deficit score, these rats (n=5) were anesthetized and euthanized in accordance with ethical guidelines through the administration of 500 mg/kg 10 % potassium chloride solution via intracardial injection, followed by rapid decapitation as a method of sacrifice. Their brains were promptly fixed in 4 % paraformaldehyde and sectioned into slices with a thickness of 3 mm, cross-cutting coronally at the optic level. These sections underwent dehydration using a gradient of alcohol, clarification with xylene, embedding in paraffin, and subsequent slicing into thin 3-μm sections, all of which were then mounted on glass slides for hematoxylin and eosin (H&E) staining. The H&E stained slides were then examined using a light microscope (Zeiss, Oberkochen, Germany), focusing on cell damage assessment. For this purpose, five non-overlapping visual fields within the ischemic cortex were randomly chosen for the identification of damaged cells showing characteristics such as vacuolar degeneration, eosinophilic degeneration, nuclear contraction, and nuclear dissolution. The total number of cells and damaged cells in each high-magnification field were quantified. The rate of cell damage was determined by the formula: (number of damaged cells/total number of cells) × 100 %.

### Quantitative Real-Time Polymerase Chain Reaction (qRT-PCR) analysis

The RNA from the infarcted cortical tissue was isolated using the RNA pure rapid extraction kit (RP1201, BioTeke, China), a method that involves guanidinium isothiocyanate and a centrifugal column. Validation of both the quality and quantity of the extracted RNA was carried out using agarose gel electrophoresis and a NanoDrop 2000 spectrophotometer (Thermo, U.S.A.) set at 260/280 nm. Subsequent to RNA isolation, the super RT kit (PR6601, BioTeke, China) employing the Moloney Murine Leukemia Virus M-MuLV method was used for first-strand complementary DNA (cDNA) synthesis with incubation conditions at 42 °C for 50 minutes followed by 70 °C for 10 minutes. The cDNA was then subject to PCR amplification in a 20 μL volume, utilizing the 2 × SYBR real-time PCR premixture kit (PR7002, BioTeke, China) in a 3-step RT-PCR protocol: an initial step at 95 °C for 2 minutes (1 cycle), followed by 45 cycles of denaturation at 95 °C for 20 seconds, annealing at 56 °C for 15 seconds, and extension at 72 °C for 20 seconds. Primers for SIRT1, Nrf2, and HO-1 were custom-designed and synthesized by Sangon Biotech (China) with specific sequences: SIRT1 (Forward: 5′-GCTGACGACTTCGACGACG-3′; Reverse: 5′-TCGGTCAACAGGAGGTTGTCT-3′), Nrf2 (Forward: 5′-TCTTGGAGTAAGTCGAGAAGTGT-3′; Reverse: 5′-GTTGAAACTGAGCGAAAAAGGC-3′), and HO-1 (Forward: 5′-AAGCCGAGAATGCTGA GTTCA-3′; Reverse: 5′-GCCGTGTAGATATGGTA CAAGGA-3′). The iCycler Thermal Cycler 96-Well Thermal Sealing Ring (iCycler iQ, Bio-Rad, U.S.A.) was employed for the PCR process, and the relative mRNA expression levels were calculated using the formula: 2^−ΔΔCT^.

### Western blot analysis

Proteins were isolated from brain tissues using a commercially available kit (Beyotime, China) for immunoblotting. After centrifugation, the supernatant containing protein samples was gathered, and their concentrations were determined using the bicinchoninic acid protein assay (BCA, Beyotime, China). Subsequently, the protein samples from each experimental group underwent separation and transfer onto nitrocellulose membranes (Millipore) *via* 10 % SDS-PAGE. To prevent nonspecific binding, the membranes were treated with a blocking solution containing 5 % nonfat dried milk for 1 hour. Primary antibodies against SIRT1 (at a 1:1000 dilution; Abcam, USA), Nrf2 (at a 1:1000 dilution; Abcam, USA), HO-1 (at a 1:1000 dilution; Sigma, USA), and β-actin as a loading control (at a 1:1000 dilution; Sigma, USA) were then applied to the membrane and left to bind overnight at 4°C. Following removal of unbound primary antibodies through washing, the membranes were exposed to the corresponding horseradish peroxidase-labeled secondary antibody (at a 1:5000 dilution, Abcam, USA) at room temperature for 1 hour. Subsequently, after further washing, the detection of antigen-antibody complexes was accomplished using an enhanced chemiluminescence solution (Bio-Rad Laboratories, USA) and quantified with Image J software.

### Biochemical analysis

Biochemical assessments were performed to analyze the enzymatic antioxidant glutathione (GSH) and superoxide dismutase (SOD) activities, as well as determine the levels of malondialdehyde (MDA), an indicator of lipid peroxidation. An enzyme-linked immunosorbent assay (ELISA) technique was employed for these measurements utilizing specific assay kits (Randox Laboratories, Ltd) according to the manufacturer’s instructions.

### Statistical analyses

The data analysis was carried out utilizing GraphPad Prism software (GraphPad Software, version 9.0, San Diego, CA, USA) designed for Windows. The results presented in this study were expressed as mean ± standard deviation (SD). An examination by one-way analysis of variance (ANOVA) and subsequent Tukey post hoc test was performed to evaluate the data. Statistical significance was considered when the P-value was below 0.05 (P < 0.05).

## Results

### Infarct volume

**Fig. 1:**
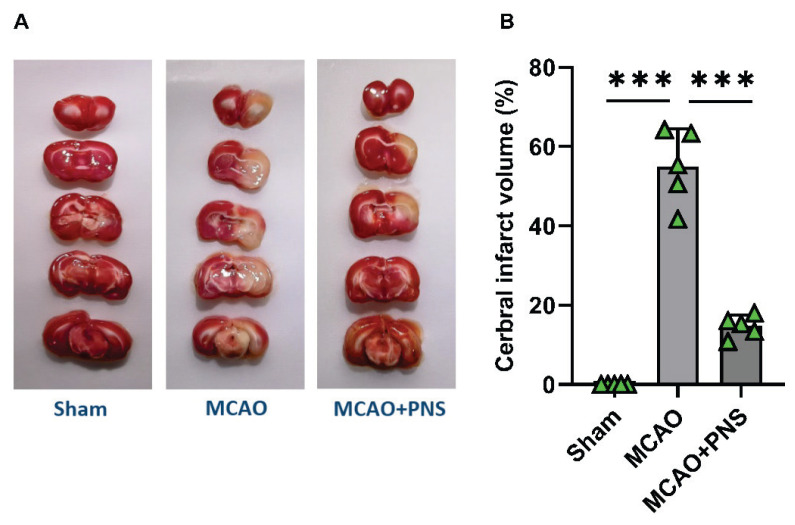
Infarct volume in different groups. TTC-stained brain slices **(A)** and statistical bar chart of determined cerebral-infarct volumes in experimental groups **(B)**. Data were analyzed using one-way ANOVA followed by Tukey’s post hoc test, and expressed as mean ± SD (n=5 per group). (***P < 0.001). MCAO: Middle cerebral artery occlusion; PNS: Panax notoginseng saponins.

In this study, we assessed the impact of PNS on cerebral infarct volume as part of exploring its potential neuroprotective properties in the setting of cerebral IR injury induced by MCAO (Fig. 1 A, B). As shown in Fig. 1 B, the MCAO model significantly increased the infarct volume to 55.05 ± 9.37 % compared to the Sham group (P < 0.001). This signifies the successful induction of cerebral IR injury in the model system. Conversely, pretreatment with PNS notably reduced the infarct volume in comparison to the IR group (P < 0.001). The mean infarct volume in the PNS-treated group was 14.75 ± 2.74 %, while the IR group exhibited a mean infarct volume of 55.05 ± 9.37 % (Fig. 1 B). Results indicate that pretreatment with PNS effectively mitigated the infarct volume in the context of MCAO-induced cerebral IR injury, demonstrating its potential neuroprotective effects within this model system.

### Neurological function

**Fig. 2:**
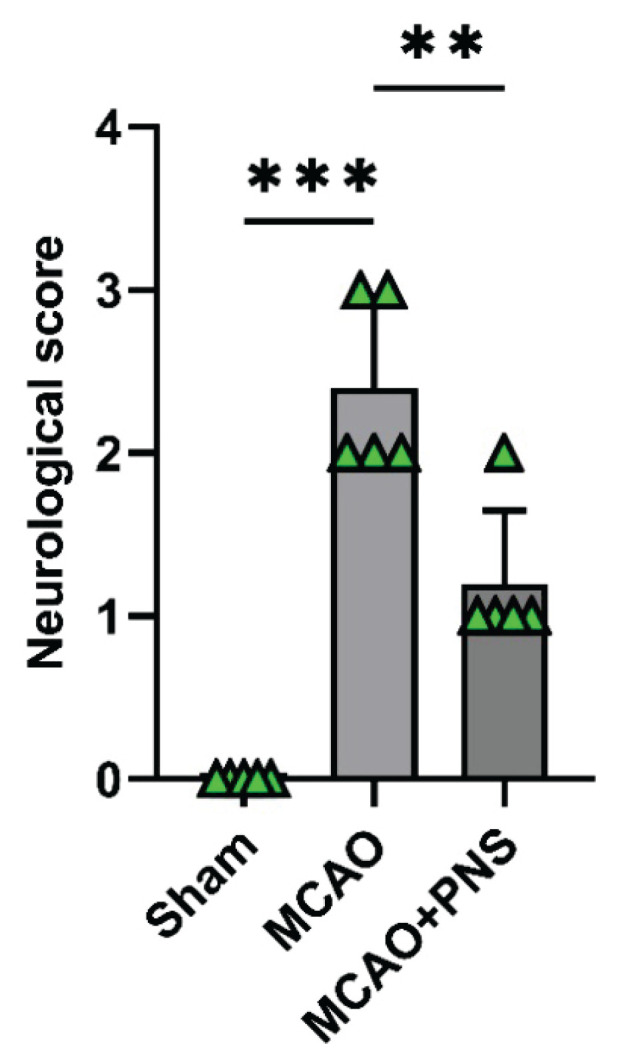
Neurological function in different groups. Statistical bar chart of evaluated neurological deficit score in experimental groups. Data were analyzed using one-way ANOVA followed by Tukey’s post hoc test, and expressed as mean ± SD (n=5 per group). (**P < 0.01 and ***P < 0.001). MCAO: Middle cerebral artery occlusion; PNS: Panax notoginseng saponins.

The effects of PNS on neurological function in rats with MCAO-induced cerebral IR injury was evaluated in this study. The Longa method was utilized to assess neurological function in the experimental animals. As depicted in Fig. 2, following the induction of MCAO, the neurological score was significantly increased compared to the Sham group (P < 0.001). This suggests the successful induction of cerebral IR injury in the model system. However, the rats in the PNS pretreatment group exhibited less severe neurological deficits compared with the rats in model group (P < 0.01). This improvement in neurological function in the PNS pretreatment group indicates a potential neuroprotective effect of PNS in the context of cerebral IR injury.

### Histopathological changes

**Fig. 3:**
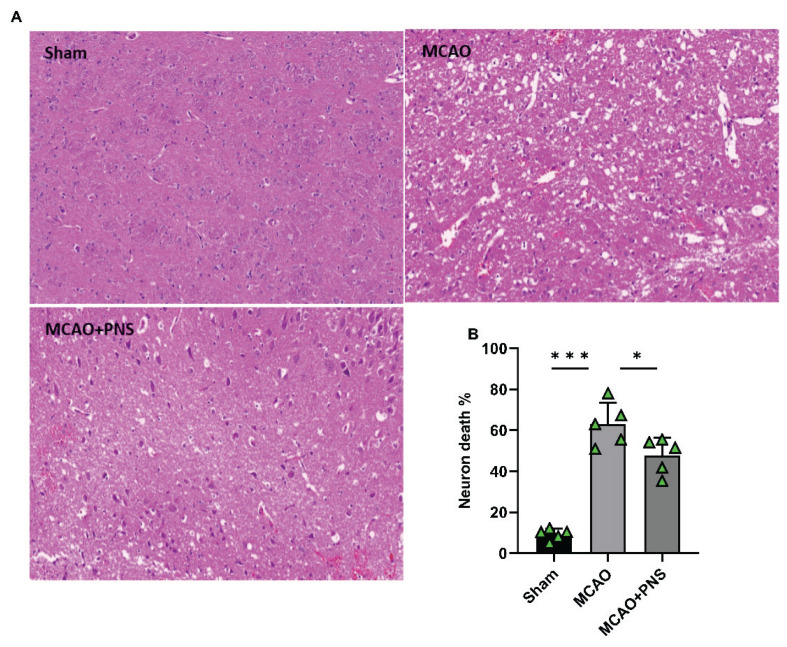
Histopathological changes in different groups. Pathological changes in the cerebral cortex evaluated by hematoxylin and eosin staining (**A)** and statistical map of the cell injury rate in each group **(B)**. Data were analyzed using one-way ANOVA followed by Tukey’s post hoc test, and expressed as mean ± SD (n=5 per group). (*P < 0.05 and ***P < 0.001). MCAO: Middle cerebral artery occlusion; PNS: Panax notoginseng saponins.

In this study, histological examination was conducted to assess the structural integrity of brain tissue and neurons in different experimental groups. As depicted in Fig. 3 A, the structure of the brain tissue and neurons was observed to be normal and intact in the Sham group. Following MCAO induction, histological examination revealed necrotic neurons, inflammatory cell infiltration, and edema around neurons and blood vessels. Additionally, vacuolar softening structures appeared in the intercellular substance. Severe nuclear pyknosis and deeper nuclear staining were observed 24 hours post-modeling, indicating increased neuronal necrosis and cellular damage. However, PNS pretreatment ameliorated ischemic morphological changes, such as cellular swelling, nucleus atrophy, and bubble-like intercellular substances compared to the MCAO group (Fig. 3 A). Fig. 3 B shows the number of damaged cells in each high-magnification field. As shown, the rate of cell damage was significantly increased following MCAO modeling compared to the Sham group (P < 0.001) (Fig. 3 B). However, PNS pretreatment led to a significant decrease in the number of damaged cells compared to the MCAO group (P < 0.05) (Fig. 3 B). In conclusion, the histopathological findings suggest that PNS pretreatment mitigates neuronal damage and structural changes induced by MCAO-induced cerebral IR injury.

### Gene expression

**Fig. 4:**
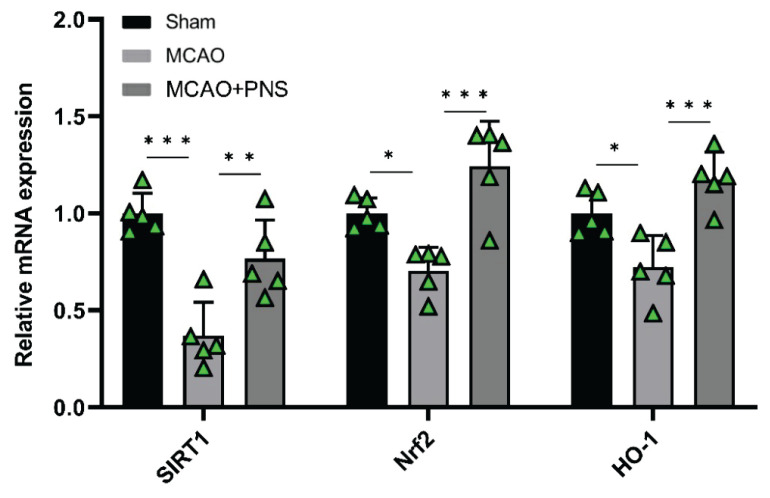
Gene expression in different groups. The expression levels of sirtuin 1 (SIRT1), nuclear factor erythroid 2-related factor 2 (Nrf2), and heme oxygenase-1 (HO-1) genes in brain tissue of experimental groups. Data were analyzed using one-way ANOVA followed by Tukey’s post hoc test, and expressed as mean ± SD (n=5 per group). (*P < 0.05, **P < 0.01, and ***P < 0.001). MCAO: Middle cerebral artery occlusion; PNS: Panax notoginseng saponins.

The gene expression levels of SIRT1, Nrf2, and HO-1 in brain tissues were evaluated to understand how these genes are affected by ischemic injury and whether a potential therapeutic agent, in this case, PNS, can modulate their expression to provide neuroprotective effects. As shown in Fig. 4, the induction of the MCAO model led to a significant decrease in the expression levels of SIRT1, Nrf2, and HO-1 genes compared to the Sham group (P < 0.001, P < 0.05, and P < 0.05, respecttively). On the other hand, pretreatment with PNS significantly increased the expression levels of SIRT1, Nrf2, and HO-1 genes compared to the MCAO group (P < 0.01, P < 0.001, and P < 0.001, respectively) (Fig. 4). Conclusively, the gene expression analysis suggests that PNS pretreatment has a notable effect on upregulating the expression of SIRT1, Nrf2, and HO-1 genes in the context of ischemic injury induced by the MCAO model. These findings highlight the potential neuroprotective properties of PNS and warrant further investigation into its therapeutic potential in ischemic conditions.

### Protein expression

**Fig. 5:**
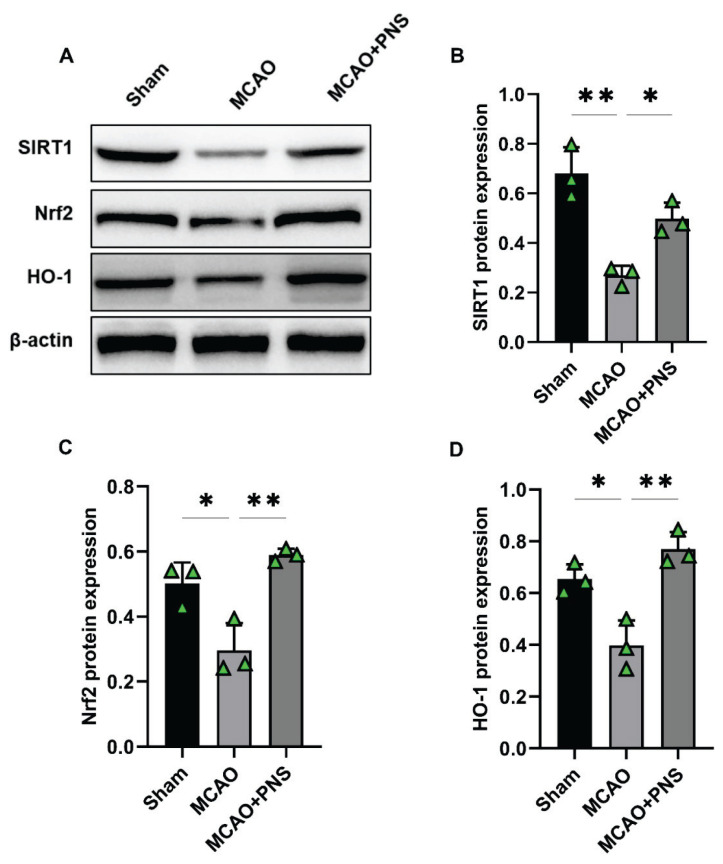
Protein expression in different groups. Representative immunoblots **(A)** and densitometry scanning of band densities to quantify the expression levels of sirtuin 1 (SIRT1) **(B)**, nuclear factor erythroid 2-related factor 2 (Nrf2) **(C)**, and heme oxygenase-1 (HO-1) **(D)** in brain tissue of experimental groups. Data were analyzed using one-way ANOVA followed by Tukey’s post hoc test, and expressed as mean ± SD (n=5 per group). (*P < 0.05 and **P < 0.01). MCAO: Middle cerebral artery occlusion; PNS: Panax notoginseng saponins.

Western blotting analysis was used to assess the protein expression levels of SIRT1, Nrf2, and HO-1 in brain tissues to determine the effects of PNS on key proteins involved in oxidative stress and cellular damage (Fig. 5 A). As shown in Fig. 5 B-D, the induction of the MCAO model led to a significant decrease in the expression levels of SIRT1, Nrf2, and HO-1 proteins compared to the Sham group (P < 0.01, P < 0.05, and P < 0.05, respectively). However, pretreatment with PNS led to a significant increase in the expression levels of SIRT1, Nrf2, and HO-1 proteins compared to the MCAO group (P < 0.05, P < 0.01, and P < 0.01, respectively) (Fig. 5 B–D). These findings suggest that PNS plays a role in enhancing the protein expression of SIRT1, Nrf2, and HO-1, indicating its potential to activate the SIRT1/Nrf2 signaling pathway. This activation may contribute to the neuroprotective benefits of PNS in the context of cerebral IR injury.

### Levels of oxidative stress markers

**Fig. 6:**
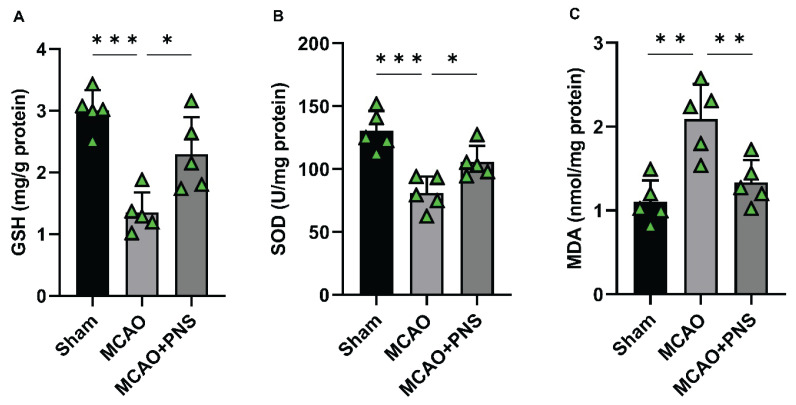
Changes of oxidative stress markers in different groups. The activities of glutathione (GSH) **(A)** and superoxide dismutase (SOD) **(B)**, and the content of malondialdehyde (MDA) **(C)** in brain tissue of experimental groups. Data were analyzed using one-way ANOVA followed by Tukey’s post hoc test, and expressed as mean ± SD (n=5 per group). (*P < 0.05, **P < 0.01, and ***P < 0.001). MCAO: Middle cerebral artery occlusion; PNS: Panax notoginseng saponins.

Oxidative stress plays a pivotal role in the pathophysiology of cerebral IR injury. In this context, the activities of enzymatic antioxidants, such as GSH and SOD, and the levels of MDA, a marker of lipid peroxidation, are key indicators of oxidative stress. Examining these oxidative stress markers provides insight into the redox imbalance and cellular damage associated with cerebral IR injury. The current study aimed to evaluate the effects of PNS on oxidative stress modulation in rats subjected to MCAO-induced cerebral IR injury. As shown in Fig. 6 A–C, the activities of enzymatic antioxidant GSH and SOD were significantly decreased in the MCAO group compared to the Sham group (P < 0.001 for both), while MDA levels were increased (P < 0.01) (Fig. 6 A–C). These results indicate a state of heightened oxidative stress and lipid peroxidation following MCAO-induced IR injury. Pretreatment with PNS led to elevated GSH and SOD levels compared to the MCAO group (P < 0.05 for both) (Fig. 6 A, B), suggesting an enhancement in antioxidant defense mechanisms. Furthermore, PNS pretreatment significantly reduced MDA levels compared to the MCAO group (P < 0.01) (Fig. 6 C). This reduction in lipid peroxidation levels indicates a protective effect of PNS against oxidative damage associated with cerebral ischemic injury. Conclusively, the findings demonstrate that PNS pretreatment effectively mitigates oxidative stress by enhancing enzymatic antioxidant activities while reducing lipid peroxidation levels in the context of MCAO-induced cerebral IR injury. These results highlight the potential therapeutic benefits of PNS in combating oxidative damage and promoting neuroprotection in ischemic conditions.

## Discussion

The primary goal of this study was to explore the potential protective effects of administering PNS to rats before inducing cerebral IR injury, and to gain insights into the underlying mechanisms. The study’s findings revealed that pretreatment with PNS notably decreased the infarct volume and neuronal cell loss, while simultaneously improving neurological function in rats following cerebral IR injury. Notably, PNS pretreatment led to a significant increase in the expression of SIRT1, Nrf2, and HO-1 genes and proteins, and also reduced the oxidative stress induced by IR in the brain. These positive effects of PNS were found to be linked, at least in part, to the regulation of the SIRT1/Nrf2/HO-1 signaling pathway.

The collective body of research consistently underscores the therapeutic benefits of PNS across various animal models, attributing these effects to the diverse pharmacological properties exhibited by PNS, such as antioxidant, anti-inflammatory, and anti-apoptotic activities [16,32,33]. PNS have demonstrated the capacity to scavenge free radicals and counteract oxidative stress, a pivotal factor implicated in neuronal damage and the progression of neurodegenerative diseases. By reducing oxidative stress levels, PNS may help preserve neuronal integrity and promote neuronal survival under challenging conditions [17, 34, 35]. Moreover, the anti-inflammatory attributes of PNS play a critical role in mitigating inflammation-induced brain damage, particularly evident in conditions like cerebral IR injury where inflammation significantly exacerbates neuronal injury. By modulating the inflammatory response, PNS effectively shield neurons from the detrimental effects of inflammation [36]. Notably, PNS have exhibited the ability to inhibit apoptosis, the programmed cell death process contributing to neuronal loss in diverse neurological disorders. By impeding apoptosis, PNS serve as sentinels against unnecessary neuronal demise, thereby bolstering neuronal survival [37]. The multifaceted neuroprotective effects of PNS encapsulate a spectrum of mechanisms, including antioxidant, anti-inflammatory, anti-apoptotic actions, and modulation of signaling pathways. These combined properties position PNS as a promising candidate for the development of therapeutic strategies aimed at safeguarding and conserving neuronal function in a range of neurodegenerative conditions [16,38,39]. The current study contributes to this growing body of knowledge by further elucidating the neuroprotective impact of PNS in the context of cerebral IR injury in rats. Our findings align with prior research, showcasing that PNS pretreatment effectively reduced infarct volume and neuronal loss, and enhanced neurological function in rats subjected to cerebral IR injury. Subsequent investigations are warranted to explore the therapeutic potential of PNS in clinical applications and unveil its comprehensive effects on neurological outcomes in ischemic scenarios.

In this study, we delved into the intricate molecular pathways underpinning the neuroprotective effects of PNS in a rat model of cerebral IR injury. Our focus centered on unraveling the intricate involvement of the SIRT1/Nrf2/HO-1 signaling pathway and its influence on antioxidant enzymes and markers of oxidative stress, including GSH, SOD, and MDA, in mediating the beneficial impacts of PNS on neuronal survival and the protection of brain tissue during ischemic injury. Cerebral IR injury results in a cascade of events that contribute to neuronal damage and cell death [7]. One of the key mechanisms underlying neuronal injury in cerebral IR is oxidative stress [8]. During ischemia, the deprivation of oxygen and nutrients leads to an imbalance in ROS production and antioxidant defense mechanisms, resulting in oxidative stress. Upon reperfusion, the sudden reintroduction of oxygen exacerbates ROS generation, leading to oxidative damage to cellular components such as lipids, proteins, and DNA, ultimately contributing to neuronal injury and cell death [40,41]. In response to oxidative stress, cells activate various signaling pathways to mitigate damage and promote cell survival. Among these pathways, the SIRT1/Nrf2/HO-1 signaling cascade plays a pivotal role in orchestrating antioxidant responses. SIRT1 can modulate Nrf2 activity by deacetylating it, thereby promoting its translocation into the nucleus and subsequent activation of antioxidant response genes, including HO-1. HO-1, a potent antioxidant enzyme, is instrumental in protecting cells from oxidative stress by catalyzing the degradation of heme and generating antioxidant molecules [12, 13, 42]. In this study, the findings revealed that pretreatment with PNS significantly reduced oxidative stress markers such as MDA and enhanced enzymatic antioxidant activities like GSH and SOD. This indicates that PNS exerts potent antioxidative effects, potentially by activating the SIRT1/Nrf2/HO-1 pathway, as evidenced by the upregulation of these genes and proteins following PNS administration. Overall, these results suggest that PNS confers neuroprotection against cerebral IR injury by attenuating oxidative stress through the modulation of the SIRT1/Nrf2/HO-1 signaling pathway. By enhancing antioxidant defenses and reducing oxidative damage, PNS shows potential as a neuroprotective agent within the context of traditional Chinese medicine. Further research into the precise mechanisms underlying the relationship between oxidative stress and the SIRT1/Nrf2/HO-1 pathway in PNS-mediated neuroprotection is warranted to better understand its potential role as a complementary intervention for cerebral IR injury.

## Limitations and suggestions

Standardizing PNS extracts to ensure consistent concentrations of active ingredients is essential for reliable results across studies. Future research should consider factors such as harvest period, geographic location, and cultivation conditions, as these can influence the biochemical composition of Panax notoginseng and impact the extract’s efficacy. This will help accurately assess the therapeutic potential of PNS extracts and their clinical applications. This study primarily focused on PNS as a singular therapeutic agent, without investigating potential synergistic interactions with other neuroprotective or antioxidant strategies. Future research should explore the combined effects of PNS with other therapeutic approaches, such as mitochondrial transplantation, stem cell therapy, or conventional treatments, to better understand their potential interactions and optimize strategies for managing cerebral IR injury [43–45]. Understanding these synergies could lead to more effective therapeutic strategies for managing cerebral IR injury and its associated neurological impairments. Additionally, expanding the scope of research to include the effects of PNS on other relevant pathways, such as autophagy [46] and inflammatory cascades [47] would provide a more comprehensive understanding of its neuroprotective mechanisms. Moreover, while our study focused on pretreatment, exploring the efficacy of PNS as a post-treatment following ischemic injury would more closely reflect clinical realities, where interventions are typically administered after the event. Investigating the potential of PNS in post-treatment models could provide important insights into its therapeutic applications for patients after ischemic events have occurred. Finally, integrating comprehensive behavioral and functional assessments in future studies would offer a more holistic view of the neuroprotective effects of PNS, enabling a better evaluation of cognitive and neurological outcomes alongside molecular changes.

## Conclusion

In conclusion, the findings from this study shed light on the neuroprotective effects of PNS in the context of cerebral IR injury. Through the activation of the SIRT1/Nrf2/HO-1 signaling pathway, PNS demonstrated potent antioxidant properties and enhanced cellular resilience in response to oxidative stress induced by IR injury. The increase in key antioxidant enzymes such as GSH and SOD, coupled with the decrease in MDA levels, further supports the ability of PNS to mitigate oxidative damage and maintain cellular integrity in the brain. The comprehensive understanding of the molecular mechanisms underlying the neuroprotective effects of PNS offers valuable insights into its therapeutic potential for combating cerebral IR injury. However, further research is needed to explore the translational relevance of these findings to human clinical applications, including clinical trials and studies involving human subjects. By addressing the limitations and suggestions outlined in this study, future research efforts can expand on current knowledge and advance the development of PNS-based therapies for ischemic conditions. By unraveling the intricate interplay between PNS, antioxidant pathways, and neuroprotection, we pave the way for innovative therapeutic strategies with the potential to alleviate oxidative stress-related damage in the brain and enhance outcomes for patients affected by cerebral IR injury.
